# Invasive micropapillary carcinoma of the breast had no difference in prognosis compared with invasive ductal carcinoma: a propensity-matched analysis

**DOI:** 10.1038/s41598-018-36362-8

**Published:** 2019-01-22

**Authors:** Shuang Hao, Yuan-Yuan Zhao, Jin-Juan Peng, Fei Ren, Wen-Tao Yang, Ke-Da Yu, Zhi-Ming Shao

**Affiliations:** 1 Department of Breast Surgery, Cancer Center and Cancer Institute, Shanghai Medical College, Fudan University, 270 Dong-An Road, Shanghai, 200032 P. R. China; 2 Department of Oncology, Shanghai Medical College, Fudan University, 270 Dong-An Road, Shanghai, 200032 P. R. China; 3 Department of Breast Surgery, the Fourth People’s Hospital of Zhenjiang, Jiangsu, 212001 P. R. China; 4 Department of Pathology, Cancer Center and Cancer Institute, Shanghai Medical College, Fudan University, 270 Dong-An Road, Shanghai, 200032 P. R. China; 5 Institute of Biomedical Science, Fudan University, Shanghai, P.R. China

## Abstract

Invasive micropapillary carcinoma (IMPC) is a rare histopathological variant of breast carcinoma that is usually associated with poor clinical characteristics. Whether IMPC has worse prognosis than invasive ductal carcinoma (IDC) is controversial. This retrospective study examined the prognostic difference between IMPC and IDC. We analysed 327 cases of IMPC patients and 4979 IDC cases who underwent primary resection in our institution between 2008 and 2012. Using propensity score matching, the two groups were matched at 1:1 by age, tumour size, nodal status, hormone status, and HER2 status. Differences in prognosis were assessed by Kaplan-Meier estimates and Cox regression analysis. We established the IMPC group and identified 324 IDC patients by propensity score matching. The survival analysis indicated that IMPC patients had no significant reduced overall survival (*p* = 0.752) or disease-free survival (*p* = 0.578) compared with IDC patients. Multivariate Cox regression analysis revealed that IMPC was not an independent prognostic factor for disease-free survival (hazard ratio [HR] = 0.944; 95% confidential interval [CI], 0.601–1.481) or overall survival (HR = 0.727; 95% CI, 0.358–1.478). Survival analysis demonstrated no statistically significant difference between IMPC and IDC, indicating that proactive or radical clinical therapy is unnecessary.

## Introduction

Invasive micropapillary carcinoma (IMPC) is a distinct rare histopathological variant of breast cancer, first described by Siriaunkgul and Tavasoli in 1993^1^ and accounts for 3% to 6% of all invasive breast cancers^2^. It is characterized by a pseudopapillary arrangement of morule-like tumour cell clusters with reverse polarity floating in empty stromal space^3,4^, which is considered an “inside-out” growth pattern. In spite of the low incidence of IMPC, it shows a high propensity for lymphovascular invasion (LVI) and lymph node metastasis (LNM) compared with invasive ductal carcinoma (IDC)^5–8^. LVI and LNM have been associated with low expression of CD44 and high expression of vascular endothelial growth factor-C (VEGF-C)^9–12^. Wang *et al*. demonstrated that the loss of leucine zipper putative tumour suppressor 1 (LZTS1) expression was associated with LNM in IMPC patients, and LZTS1 promoter methylation could be responsible for the loss of LZTS1 expression^13^. However, whether the worse molecular expression and/or clinicopathological features may lead to poorer prognosis of IMPC remains controversial.

Previously published retrospective studies have examined prognosis in IMPC, however these were performed in a small sample of IMPC patients, and therefore the outcome has not been appropriately investigated. In this study, we analysed 327 IMPC cases and 4979 IDC cases to evaluate differences in prognosis using propensity score matching (PSM) to remove confounding factors. We then performed survival analyses of the matched IMPC and IDC groups, with the aim of applying these findings to inform therapeutic strategy.

## Methods

### Patients

We reviewed the clinical data of 327 patients with IMPC who were diagnosed and treated in the Department of Breast Surgery of the Fudan University Shanghai Cancer Center (FUSCC) between January 2008 and October 2012. All IMPC cases in the study demonstrated a micropapillary component that was in accordance with the morphological criteria described in the WHO histological classification of breast tumours^2^. We included a total number of 4979 cases of IDC as controls, as previous studies showed that a ratio of controls to cases greater than 4:1 will improve the power of the study^14^. All patients were female, and those with distant metastasis were excluded. Patients were evaluated with comprehensive physical, laboratory and image examinations prior to any therapy according to clinical practice guideline standards; evaluations included ECG, chest X-ray, bilateral mammography and ultrasonography of the breasts, axillary fossae, abdomen and pelvis region. The surgical approach and therapeutic regimen was in accordance with the National Comprehensive Cancer Network guidelines.

Approval of this study was obtained from the independent ethical committee/institutional review board of FUSCC (Shanghai Cancer Center Ethical Committee), and informed consent was obtained from all patients before the study began. All methods were performed in accordance with the relevant guidelines and regulations.

Follow-up information of tumour recurrence and survival status was acquired through outpatient departmental records and personal contact with the patients via telephone calls. The follow-ups were carried out every 3 months during the first 2 years, every 6 months during the next 2 years and once a year thereafter.

The datasets analysed during the current study are available from the corresponding author on request.

### Pathological diagnosis

Haematoxylin and eosin (H&E)-stained formalin-fixed paraffin-embedded sections from the database were reassessed by two senior pathologists. In cases of divergent opinions, the results were discussed until an agreement was reached. The IMPC specimens were classified into three groups according to the amount of IMPC present in each tumour: tumours with less than 25%, tumours with 25% to 74% and tumours with greater than 75% IMPC. Marchio stated that mixed IMPCs had similar patterns of phenotype compared with pure IMPCs, and the authors proved that mixed IMPCs were more closely related to pure IMPCs than to IDC of no special types^15^. In our study, mixed IMPCs were included into the IMPC group.

ER (oestrogen receptor), PR (progestin receptor) and HER2 (epidermal growth factor receptor 2) statuses were determined on representative paraffin sections from each tumour using immunohistochemical (IHC) staining. ER and PR expression was considered positive in cases if the number of ER- or PR-positive nuclei was greater than 1%, and cases with ER+ and/or PR+ were considered hormone receptor (HR) positive^16^. HER2 expression was evaluated using a monoclonal antibody and a peroxidase-antiperoxidase technique. The results were scored from 0 to 3+ according to the criteria of the Hercep Test^17^. Fluorescence *in situ* hybridization (FISH) tests were used when the IHC results were ambiguous (i.e., 2+), or for patients who could not be defined as HER2−. In our study, tumours with an IHC score of 3+ or with amplification confirmed by FISH were defined as HER2-positive. The pathological and IHC studies were performed using an Olympus light microscope with ×10 and ×40 magnifications by the two previously mentioned independent pathologists in the Department of Pathology of FUSCC.

### PSM

PSM is a method for filtrating experimental and control cases of similar characteristics, which are called the matching variables, from existing data to make them comparable in a retrospective analysis^18^. PSM can be used to adjust for baseline characteristics and reduce the effect of selection bias. Each patient in the study was assessed by a score calculated by potential confounders, and the two cohorts were matched due to these scores. The comparison of outcomes between two groups can be fair and avoid bias to some extent. The variables for propensity score matching were selected as follows: age (years), tumour size (cm), nodal status, HR status and HER2 status. We used a ratio of 1:1 for nearest neighbour matching within 0.2 standard deviation of the logit of the propensity score^19^.

### Statistical analysis

Primary outcome variables included disease-free survival (DFS) and overall survival (OS). DFS was defined as the time from surgery to the first date of local recurrence, regional recurrence, distant recurrence, second breast cancer or death from any cause, whichever occurred first. OS was defined as the time from surgery to death due to any cause. Survival analyses were estimated using the Kaplan-Meier method and compared using log-rank test. Univariate and multivariate analyses were calculated using the Cox risk proportion model. Hazard ratios (HR) were presented with 95% confidence intervals (CI). All comparisons were two-tailed, and *p* < 0.05 was considered statistically significant. SPSS23.0 and Prism 6 software packages were used for survival analyses, while PSM was conducted by R (https://www.r-project.org/).

## Results

### Characteristics and survival analysis of IMPC and IDC patients before PSM

**Figure 1:**
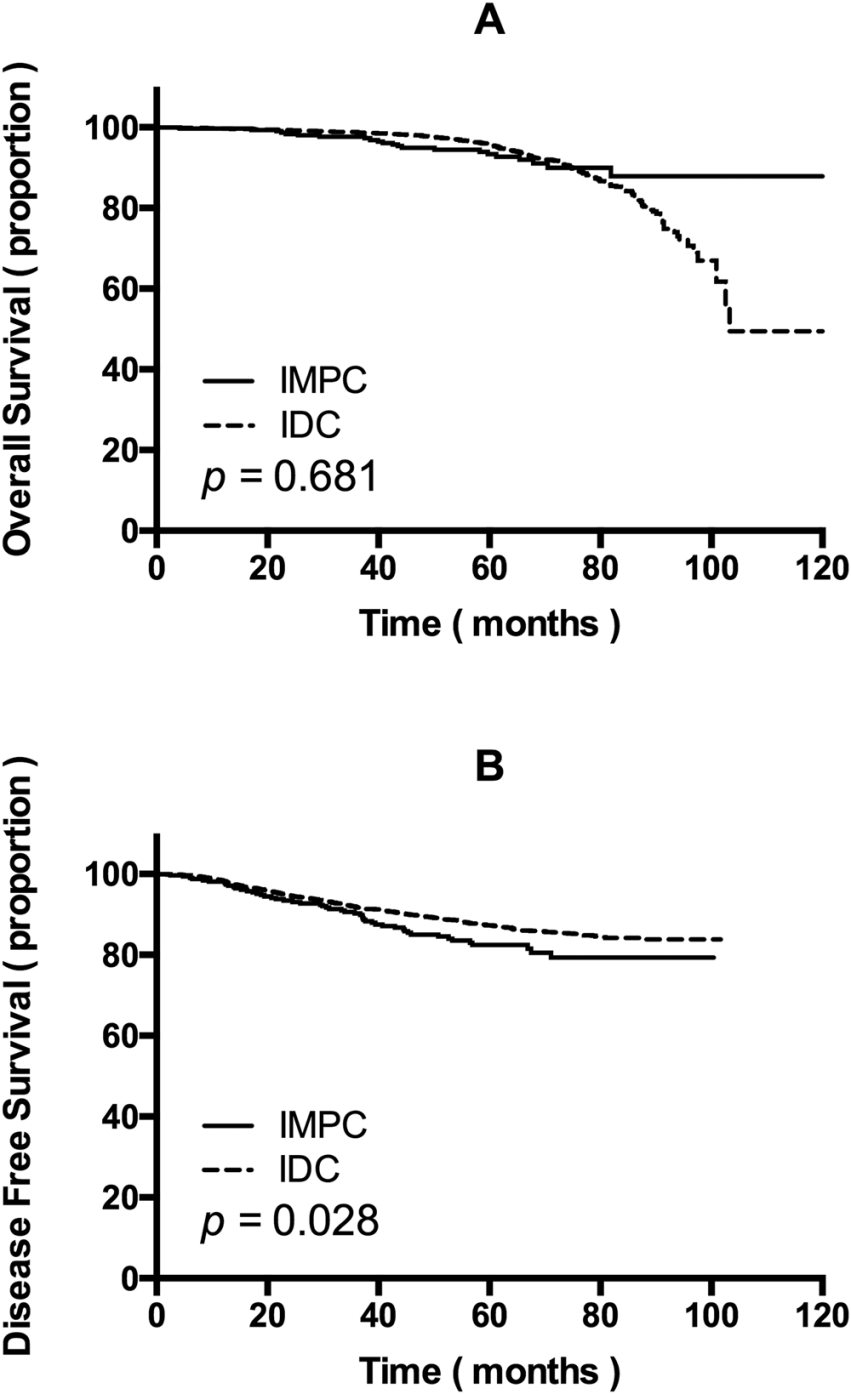
(**A**) Overall survival and (**B**) disease-free survival of invasive micropapillary carcinoma (IMPC) patients and invasive ductal carcinoma (IDC) patients before propensity score matching.

The clinical and pathological characteristics of the IMPC and IDC patients included in this study are listed in Table 1. Characteristics included age, tumour size, node status, TNM stage, ER status, PR status, HER2 status, LVI and surgical procedure. The baseline characteristics of the two groups, except for age, tumour size and surgical procedure, were statistically different. For example, the percentage of early stage disease in the IDC group was significantly higher than that in the IMPC group (stage I: 27.7% versus 17.7%; stage II: 50.2% versus 38.5%; *p* < 0.001). Albeit different, the result of survival analysis could be interfered by multiple confounding factors (Fig. 1). Because of the significant differences between these patient groups, the application of PSM to make generate comparable groups is indispensable. Finally, 324 pairs were matched from 327 IMPC patients and 4979 IDC patients; three IMPC cases were excluded because of missing data.

**Table 1:** Patient demographics and clinical characteristics before propensity score matching.

Characteristics	IMPC (n = 327) n	%	IDC (n = 4979) n	%	*p* value
**Age, years**					0.623
Mean	52.4		52.1		
SD^a^	11.0		10.7		
**Tumour size, cm**					0.121
T ≤ 2	131	40.1	2250	45.1	
2 < T ≤ 5	182	55.7	2578	51.8	
T ≥ 5	14	4.3	151	3.0	
**Node status**					0.000
0	101	30.9	2640	53.0	
1–3	85	26.0	1273	25.6	
4–9	72	22.0	623	12.5	
≥ 10	69	21.1	443	8.9	
**TNM stage**					0.000
I	58	17.7	1379	27.7	
II	126	38.5	2500	50.2	
III	143	43.7	1100	22.1	
**LVI** ^b^					0.000
yes	233	71.3	1779	35.7	
no	73	22.3	2097	42.1	
unknown	21	6.4	1103	22.2	
**Hormone status**					
ER^c^ positive	276	84.4	3743	75.2	0.000
PR^d^ positive	261	79.8	3546	71.2	0.001
HER2^e^ positive	108	33.0	1197	24.0	0.001
**Surgery**					0.292
MRM^f^	251	76.8	3836	77.0	
BCS^g^	32	9.8	599	12.0	
Mastectomy	28	8.6	315	6.4	
Others	16	4.9	229	4.6	

SD^a^: standard deviation.

LVI^b^: lymphovascular invasion.

ER^c^: oestrogen receptor.

PR^d^: progestin receptor.

HER2^e^: epidermal growth factor receptor 2.

MRM^f^: modified radical mastectomy.

BCS^g^: breast conserving surgery.

### Clinical and pathological characteristics of matched IMPC and IDC patients

The clinical and pathological characteristics of the matched IMPC and IDC patients are listed in Table 2. The mean patient age of the IMPC group was 52.4 years, while that of the IDC group was 51.7 years (*p* = 0.077). The mean tumour sizes of IMPC and IDC patients were 2.76 cm and 2.82 cm (*p* = 0.370). The mean numbers of positive lymph nodes of IMPC and IDC patients were 4.9 and 4.7 (*p* = 0.715). The clinical stage of distribution was as follows: the IMPC group: stage I, 58 patients (17.9%); stage II, 125 patients (38.3%); and stage III, 141 patients (43.8%); the IDC group: stage I, 54 patients (16.7%); stage II, 141 patients (43.5%); and stage III, 129 patients (39.8%). No significant differences were observed in the percentage of TNM stage of the two groups (*p* = 0.441) or the incidence of LVI (71.3% versus 73.1%, *p* = 0.869). Furthermore, the proportion of ER-positive patients was 84.3% in the IMPC group and 87.3% in the IDC group (*p* = 0.363), while that of PR-positive cases was 84.3% in the IMPC group and 79.6% in the IDC group (*p* = 0.845). HER2 status was positive in 33.0% of IMPCs and 30.9% of IDCs (*p* = 0.555). Patients in both groups underwent primary resection in our institution, and the surgical procedures of the two groups were not statistically different (*p* = 0.609).

**Table 2:** Patient demographic and clinical characteristics after propensity score matching.

Characteristics	IMPC (n = 324) n	%	IDC (n = 324) n	%	*p* value
**Age, years**					0.077
Mean	52.4		51.7		
SD^a^	11.0		9.7		
**Tumour size, cm**					0.370
T ≤ 2	130	40.1	126	38.9	
2 < T ≤ 5	181	55.9	177	54.6	
T ≥ 5	13	4.0	21	6.5	
**Node status**					0.715
0	101	31.2	101	31.2	
1–3	85	26.2	97	29.9	
4–9	71	21.9	66	20.4	
≥10	67	20.7	60	18.5	
**TNM stage**					0.441
I	58	17.9	54	16.7	
II	125	38.3	141	43.5	
III	141	43.8	129	39.8	
**LVI** ^b^					0.869
yes	231	71.3	237	73.1	
no	72	22.2	67	20.6	
unknown	21	6.4	20	6.2	
**Hormone status**					
ER^c^ positive	275	84.3	283	87.3	0.363
PR^d^ positive	260	84.3	258	79.6	0.845
HER2^e^ positive	107	33.0	100	30.9	0.555
**Surgery**					0.609
MRM^f^	249	76.8	263	81.2	
BCS^g^	31	9.8	25	7.7	
Mastectomy	28	8.6	23	7.1	
Others	16	4.9	13	4.0	

SD^a^: standard deviation.

LVI^b^: lymphovascular invasion.

ER^c^: oestrogen receptor.

PR^d^: progestin receptor.

HER2^e^: epidermal growth factor receptor 2.

MRM^f^: modified radical mastectomy.

BCS^g^: breast conserving surgery.

### Kaplan-Meier survival analysis of matched IMPC and IDC groups

**Figure 2:**
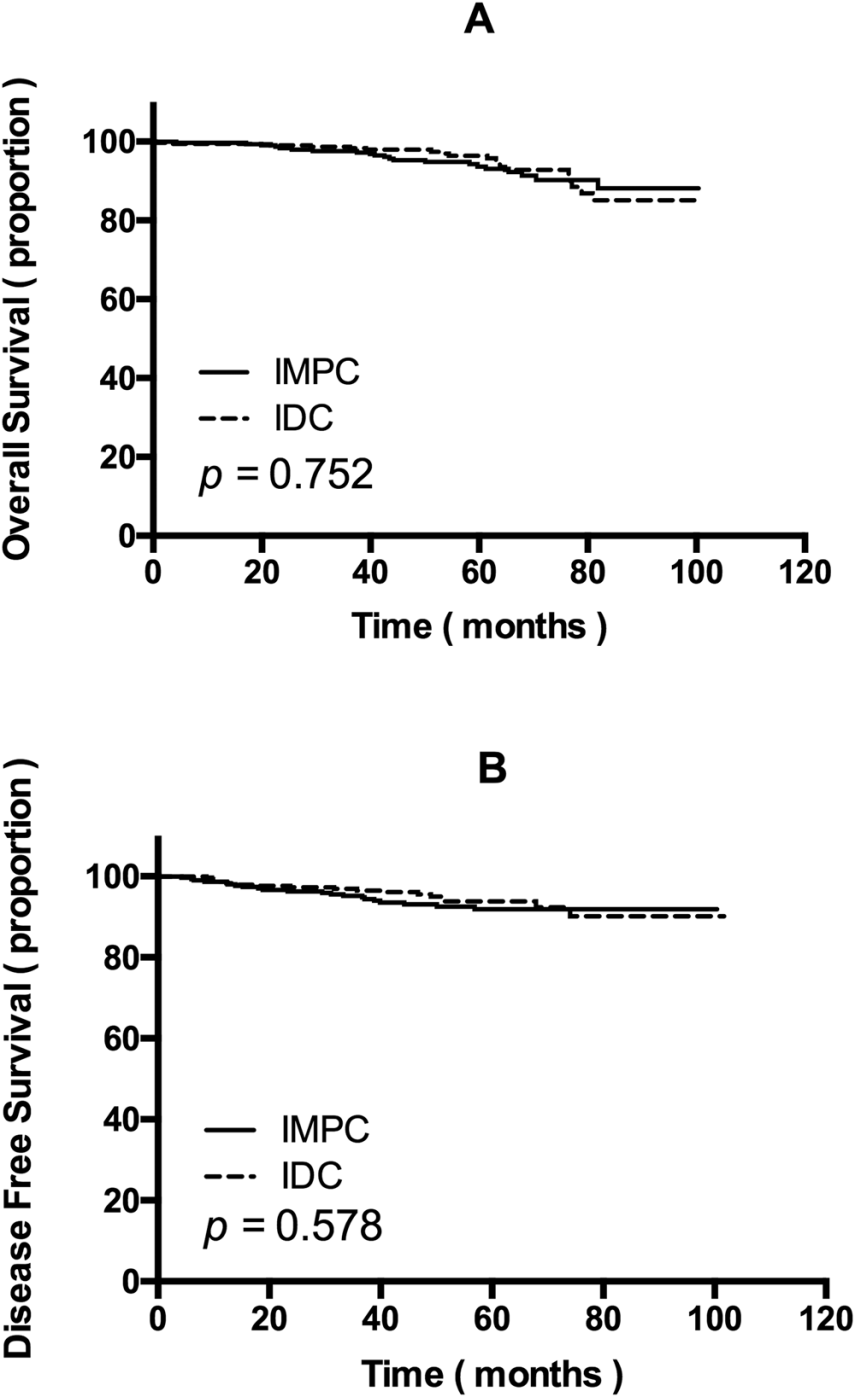
(**A**) Overall survival and (**B**) disease-free survival of invasive micropapillary carcinoma (IMPC) patients and invasive ductal carcinoma (IDC) patients after propensity score matching.

The mean follow-up of this study was 56.5 months for the IMPC group versus 51.7 months for the IDC group. At a median time of observation of 4.9 years for OS and 4.3 years for DFS, no differences were noted in locoregional or distant recurrence rates comparing IMPCs with IDCs (15.4% versus 13.6%, *p* = 0.504). Kaplan-Meier survival analysis demonstrated no statistically significant differences in DFS and OS between the two groups (Fig. 2).

### Univariate and multivariable analysis

We performed univariate and multivariate Cox regression analyses to estimate the clinical significance of prognostic factors that may affect DFS or OS of the patients. In univariate analysis, TNM stage and LVI were statistically significant for both DFS and OS (Table 3). Additionally, multivariate Cox proportional hazards regression analysis revealed that IMPC was not an independent prognostic factor for DFS (HR = 0.944; 95% CI, 0.601–1.481) or OS (HR = 0.727; 95% CI, 0.358–1.478).

**Table 3:** Univariate and multivariate analysis of overall survival and disease-free survival.

Factors		Univariate analysis		Multivariate analysis	
		HR (95% CI)	*p* value	HR (95% CI)	*p* value
**OS**					
Age	<35, 35~60, >60	1.343 (0.745–2.424)	0.327	2.737 (1.350–5.551)	0.005
Pathology	IMPC, IDC	0.905 (0.487–1.683)	0.752	0.727 (0.358–1.478)	0.379
TNM stage	I, II, III	3.590 (1.952–6.603)	<0.001	2.822 (1.362–5.850)	0.005
LVI^a^	Negative, positive	2.934 (1.133–7.601)	0.027	1.575 (0.528–4.696)	0.415
HR^b^ status	Negative, positive	0.361 (0.176–0.738)	0.005	0.277 (0.106–0.722)	0.009
HER2^c^ status	Negative, positive	1.083 (0.529–2.218)	0.827	0.833 (0.331–2.092)	0.697
**DFS**					
Age	<35, 35~60, >60	0.676 (0.457–1.001)	0.051	0.888 (0.564–1.397)	0.606
Pathology	IMPC, IDC	0.901 (0.601–1.352)	0.616	0.944 (0.601–1.481)	0.802
TNM stage	I, II, III	3.082 (2.155–4.408)	<0.001	2.665 (1.731–4.103)	<0.001
LVI	Negative, positive	2.053 (1.204–3.502)	0.008	0.960 (0.513–1.799)	0.900
HR status	Negative, positive	0.786 (0.429–1.441)	0.437	1.059 (0.517–2.167)	0.876
HER2 status	Negative, positive	1.554 (1.007–2.399)	0.046	1.314 (0.798–2.166)	0.284

LVI^a^: lymphovascular invasion.

HR^b^: hormone receptor.

HER2^c^: epidermal growth factor receptor 2.

## Discussion

Breast cancer is the most common female malignant carcinoma around the world and represents the primary cause of cancer-related death among women^20^. The reported incidence of breast cancer in China, especially in developed areas such as Shanghai, has increased significantly in recent years^21^. Breast cancer is a type of histologically heterogeneous disease that exhibits different biological behaviours and shows different pathological subtypes^22^. IMPC is a rare pathological subtype of breast cancer. A previous study indicated that most patients with IMPC had mixed IMPC, and the pure variant of IMPC is very rare^22,23^. These results are consistent with those of our study. IMPC has been linked with a high tendency of LVI and LNM and thus was considered more aggressive than IDC; IMPC has therefore been the subject of special attention in the past decades^23–30^. Until now, there has been no prospective study of IMPCs and comparative analysis between IMPC and IDC has also been rare. Therefore, whether IMPC is associated with worse prognosis and survival has been unclear.

In 2008, Chen *et al*. showed that the 5-year and 10-year survival of IMPC was significantly lower than that of IDC^25^. Later, Yu *et al*. reported that the 5-year OS and distant metastasis-free survival showed no statistically differences between IMPC and IDC groups, but the locoregional recurrence-free survival at 5 years was significantly lower in the IMPC group^26^. However, the study by Vingiani *et al*. 2013 showed that DFS and OS was not statistically different between IMPC and IDC^27^, and these findings are identical with our results but were based in a small sample of individuals. A retrospective study conducted by Liu in 2014 demonstrated that there was no difference in DFS between IMPCs and LN-matched patients^28^. In a retrospective multicentre study in 2015, Yu suggested that the distant metastasis rate and OS of the IMPC group did not differ from the IDC group, but locoregional recurrence survival and recurrence-free survival were significantly different, which emphasized the recurrence pattern of IMPC^29^. In 2015, Chen *et al*. showed that pN was the only independent prognostic factor of LRRFS and metastasis-free survival for IMPC, indicating that IMPC is featured with high rate of lymph node involvement, which is strongly associated with high rate of locoregional recurrence^30^. However, there is a lack of studies comparing the prognosis of IMPC and IDC between patients matched for age at initial diagnosis, tumour size, node status, HR status, HER2 status and LVI. So far, our study is the largest to show the prognosis of IMPC compared with IDC using PSM. Our population-based study demonstrates that although patients with IMPC tend to have advanced nodal status and higher clinical stage, the survival of IMPC patients is similar to that in IDC patients after PSM.

Here we first analysed the clinical and pathological characteristics of 327 IDC patients and 4979 IMPC patients after reviewing their data. The baseline characteristics were significantly unbalanced. IMPC patients tended to have worse nodal status, advanced clinical stage, more frequency of LVI and higher rates of positive ER, PR and HER2 status. A higher rate of LNM and LVI could give rise to lower DFS, but a higher frequency of positive ER, PR and HER2 status implicates more application of hormone and targeted therapy, which might be considered as an explanation for the similar OS but different DFS of the two groups before PSM. As it is widely accepted that the unbalanced baseline characteristics could result in interference of outcome, we used PSM to eliminate bias. After matching the remarkable differences of clinicopathological characteristics of IMPCs with IDCs, including age, tumour size, nodal status, HR status and HER2 status, we demonstrated that the OS and DFS of the two groups were not substantially different. Furthermore, multivariate Cox proportional hazards regression analysis revealed that this pathological subtype was not an independent prognostic factor for DFS and OS. Our result provides evidence that the micropapillary histotype does not add any independent information to the risk of clinical outcome and reinforces the opinion that invasive micropapillary breast carcinoma usually arises as a locally advanced disease, which is in line with several previous studies, such as the report by Yu *et al*. Based on the current results, acquisition of adequate axillary LN assessment and an appropriate approach for axillary LN management should be applied to improve clinical outcome of IMPC, and aggressive clinical therapy seems to be unnecessary. Clinical specialists should pay attention to the nodal and LVI status when determining a therapeutic strategy for a patient diagnosed with IMPC. If possible, an axillary ultrasound or even magnetic resonance imaging should be recommended before axillary management, especially for IMPC patients with small primary tumours. Meanwhile, the implement of sentinel lymph node biopsy should be prudent.

This study had several limitations. First, bias associated with the retrospective assessment of prospectively collected data may exist, although we attempted to use PSM to address this problem. Second, Ki-67 status and histological grade were not included in the current analysis, and these are considered as common prognostic factors. Third, the mean follow-up for the IMPC group was only 56.5 months, so this study may be not able to determine whether there are any differences in long-term recurrence or metastasis between the two groups.

Despite these limitations, to the best of our knowledge, this study is the largest comparative study of IMPC and IDC using PSM to examine prognosis. We believe that this study increases confidence concerning the relationship between unique clinical characteristics and treatment strategy in IMPC. A multicentre, prospective study with long-term follow-up is essential to verify the findings of our study.

## Conclusions

The results from our PSM analysis suggested that there was no statistically significant difference in prognosis between IMPC and IDC groups after matching them with similar clinical characteristics. Although IMPC appears more aggressive and has increased lymph node involvement, patients diagnosed with IMPC may not have to undergo proactive or radical clinical therapy. IMPC is not a significantly important factor that should be taken into consideration when specialists define the therapeutic procedures. Our findings provide valuable insights into the prognosis and treatment strategy of this unusual variant of invasive breast cancer.
